# A Novel Long Non-Coding RNA in the *hTERT* Promoter Region Regulates *hTERT* Expression

**DOI:** 10.3390/ncrna4010001

**Published:** 2017-12-29

**Authors:** Sanandan Malhotra, Mallory A. Freeberg, Shelby J. Winans, James Taylor, Karen L. Beemon

**Affiliations:** Department of Biology, Johns Hopkins University, Baltimore, MD 21218, USA; smalhot7@jhu.edu (S.M.); mallory.freeberg@gmail.com (M.A.F.); swinans1@jhu.edu (S.J.W.); james@taylorlab.org (J.T.)

**Keywords:** lncRNA, TERT, cancer, bidirectional transcription

## Abstract

A novel antisense transcript was identified in the human telomerase reverse transcriptase (*hTERT*) promoter region, suggesting that the *hTERT* promoter is bidirectional. This transcript, named *hTERT* antisense promoter-associated (*hTAPAS*) RNA, is a 1.6 kb long non-coding RNA. *hTAPAS* transcription is initiated 167 nucleotides upstream of the *hTERT* transcription start site and is present in both the nucleus and the cytoplasm. Surprisingly, we observed that a large fraction of the *hTERT* polyadenylated RNA is localized in the nucleus, suggesting this might be an additional means of regulating the cellular abundance of hTERT protein. Both *hTAPAS* and *hTERT* are expressed in immortalized B-cells and human embryonic stem cells but are not detected in normal somatic cells. *hTAPAS* expression inversely correlates with *hTERT* expression in different types of cancer samples. Moreover, *hTAPAS* expression is not promoted by an *hTERT* promoter mutation (-124 C>T). Antisense-oligonucleotide mediated knockdown of *hTAPAS* results in an increase in *hTERT* expression. Conversely, ectopic overexpression of *hTAPAS* down regulates *hTERT* expression, suggesting a negative role in *hTERT* gene regulation. These observations provide insights into *hTAPAS* as a novel player that negatively regulates *hTERT* expression and may be involved in telomere length homeostasis.

## 1. Introduction

Telomerase activity and telomere length have important implications in human disease and aging [1]; elevated telomerase activity has been detected in most human cancers [2,3,4]. Expression of human telomerase reverse transcriptase (*hTERT*), the enzymatic component of telomerase, is tightly regulated at the transcriptional level through epigenetic modifications in the promoter region [5], as well through alternative splicing [6,7,8]. Abundance of *hTERT* is a rate-limiting step in modulating telomerase activity [9]. While normally expressed in the germline and stem cells, up-regulated *hTERT* is essential for the continual proliferation and long-term viability of cells in many cancers [1,4]. Recurrent mutations in the *hTERT* promoter region, at -66 or -88 nucleotides (nts) relative to the *hTERT* transcriptional start site (TSS), are among the most common somatic mutations in many types of cancer, including melanomas, glioblastoma multiforme, hepatocellular carcinomas, and bladder cancers [7,10,11].

The *hTERT* promoter has many features characteristic of bidirectional promoters, such as the absence of a TATA box and high GC content [12], as well as binding sites for the ETS transcription factor GA binding protein (GABP) [13,14]. Recent data demonstrate that the mutant *hTERT* promoter can be bound and activated by GABP [15], suggesting it may induce bidirectional transcription of an antisense transcript.

We have previously reported retroviral activation of an antisense transcript upstream of *TERT* in chicken B-cell lymphomas, named *TERT* antisense promoter-associated (*TAPAS*) RNA [16]. Truncated *TAPAS* RNA is up regulated in chicken B-cell lymphomas. Here, we identify and characterize a human *TAPAS* (*hTAPAS*) RNA transcript in many types of human cancer and in several cell lines, using bioinformatics and experimental analyses. The *hTAPAS* transcript spans approximately 1.6 kb and has a single unspliced exon. Further, the absence of any conserved large open reading frames (ORFs) with protein domain homology suggests that this transcript is a previously unidentified long non-coding RNA (lncRNA). 

We observe that *hTAPAS* expression is inversely correlated with *hTERT* expression in human cancers in The Cancer Genome Atlas (TCGA). Furthermore, we do not observe any activation of *hTAPAS* expression with the *hTERT* promoter mutation in a mini-gene construct expressed in human embryonic kidney (HEK-293) cells. Knocking down of *hTAPAS* via antisense-oligonucleotides increases *hTERT* expression, and ectopic overexpression of *hTAPAS* down regulates *hTERT* expression. This suggests that *hTAPAS* is involved in negatively regulating *hTERT* expression. Our work confirms the existence of an antisense lncRNA, *hTAPAS*, upstream of *hTERT* that exhibits negative regulation of *hTERT* expression. Moreover, we observe that nearly half of the *hTERT* transcript is localized in the nucleus, suggesting this might serve as an additional way of regulating the cellular abundance of *hTERT* protein.

## 2. Results

### 2.1. An Antisense Transcript Is Expressed Upstream of hTERT

**Figure 1:**
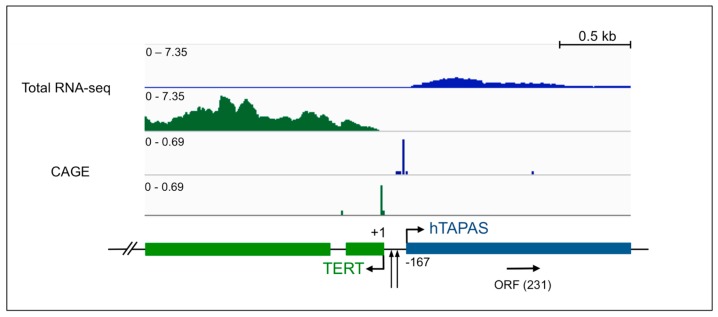
An antisense RNA, named human *TAPAS* RNA, is expressed in the human telomerase reverse transcriptase (*hTERT*) promoter region. Normalized and stranded RNA sequencing (RNA-seq) (Bedgraph) transcription coverage for *hTERT* (green) and *hTAPAS* (blue) expression, as well as corresponding cap analysis gene expression (CAGE) start sites, are depicted for the human B-cell line GM12878. The schematic below denotes Reverse Transcription PCR (RT-PCR) validation of *hTAPAS* transcript from human embryonic kidney (HEK-293) cells by tiling arrays. The first two exons of *hTERT* and an approximately 1.6 kb long *hTAPAS* gene, located 167 nts upstream of the *hTERT* transcriptional start site are depicted. Arrowheads between *hTERT* and *hTAPAS* represent sites of point mutations in the *hTERT* promoter, located 124 or 146 nucleotides upstream of the *hTERT* translational start site.

Deep sequencing of transcriptomes from chickens revealed an antisense transcript upstream of *TERT*, which is an alternatively spliced, polyadenylated, lncRNA named *TAPAS* [16]; this suggests that the *TERT* promoter is bidirectional. To determine whether a similar transcript is expressed in humans, we first examined RNA sequencing data from the Encyclopedia of DNA Elements (ENCODE) Consortium [17]. An antisense RNA in the *hTERT* promoter region was readily observed in two different human B-cell tumor lines (GM12878 and OCI-LY7) (Figure 1 and Figure S1). Moderate expression levels of this transcript were also observed in a human embryonic stem cell line (H1-hESC), and to a lesser extent in a human hepatocellular carcinoma cell line (HepG2) (Figure S1). However, this transcript was not detected in HeLa (human cervical carcinoma) and K562 (human leukemic) cell lines (Figure S1). All of these cell lines expressed *hTERT* (Figure S1). 

To better determine the 5′ end of *hTAPAS*, we used 5′ cap analysis gene expression (CAGE) data from the ENCODE Consortium for the GM12878 cell line [17]. 5′ CAGE data specifically identifies the 5′ ends of capped transcripts, indicating TSSs. We observe a strong signal starting 167 nts upstream of the annotated *hTERT* TSS and in the opposite orientation (Figure 1). Taken together, the RNA sequencing (RNA-seq) and 5′ CAGE data from the ENCODE Consortium suggest that an antisense transcript, hereafter referred to as the *hTERT* antisense promoter-associated (*hTAPAS*) RNA, was detected in the *hTERT* promoter region in human embryonic stem cells and in some but not all cancer cell lines. Interestingly, the *hTERT* promoter mutations found in several tumor types, -124 C>T or -146 C>T [7,10,11], lie in between the TSS for *hTERT* and *hTAPAS* (Figure 1).

From TCGA we downloaded normal and tumor tissue RNA-seq read alignments of approximately 3800 samples from eight different cancer types, (Table S1), and reconstructed transcripts de novo to identify annotated and novel transcripts (Figures S2 and S3A). In addition to identifying *hTERT* transcript isoforms, we identified an approximately 1.6 kb long *hTAPAS* transcript at the locus predicted by the ENCODE data, in some tumors (Figure S3A). Low expression levels were observed for both *hTAPAS* and *hTERT* in corresponding normal tissues from cancer patients (Figure S3B).

Finally, to experimentally validate expression of this transcript, we performed a tiling array Reverse Transcription PCR (RT-PCR), using RNA from HEK-293 cells, and again identified an approximately 1.6 kb long *hTAPAS* RNA (Figure 1). We also detected the *hTAPAS* transcript in HeLa and hepatocellular carcinoma (SNU-449 and SNU-475) cells via RT-PCR analysis. Based on these results and the ENCODE Consortium RNA-seq and CAGE data, we predict that *hTAPAS* is about 1.6 kb long with a single unspliced exon and is transcribed starting at 167 nts upstream of the *hTERT* TSS. 

### 2.2. hTAPAS Exhibits Features of a Long Non-Coding RNA

There is a small ORF in the middle of *hTAPAS* RNA (231 nts) (Figure 1). However, this ORF is not conserved and lacks any protein domain homology, implicating this transcript as a lncRNA. Unlike *hTERT* exons, the *hTAPAS* transcribed locus does not exhibit evolutionarily conserved elements as determined by phastCons scores (Figure S4A) [18]. Positions representing the *hTERT* exons had a mean phastCons score of 0.204, *hTAPAS* locus positions had a mean score of 0.048, and random positions had a mean score of 0.047. *hTAPAS* also shows evidence of faster evolution compared to neutral evolution rates as determined by phyloP scores (Figure S4A) [19]. phyloP scores were aggregated for *hTAPAS*, *hTERT* exons, and random positions. Positions representing *hTERT* exons had a mean phyloP score of 0.089 (positive scores indicate conservation), the *hTAPAS* locus had a mean score of −0.081, and random positions had a mean score of −0.1236 (negative scores indicate faster evolution compared to a neutral evolution rate). Thus, similar to the previously reported chicken *TAPAS* transcript, *hTAPAS* lacks ORFs with known protein domain homology and exhibits no protein-coding potential based on phyloCSF scores (Figure S4B) [20]. Based on these features, we propose the *hTAPAS* transcript to be a lncRNA.

### 2.3. Sub-Cellular Localization of hTAPAS and hTERT RNAs

**Figure 2:**
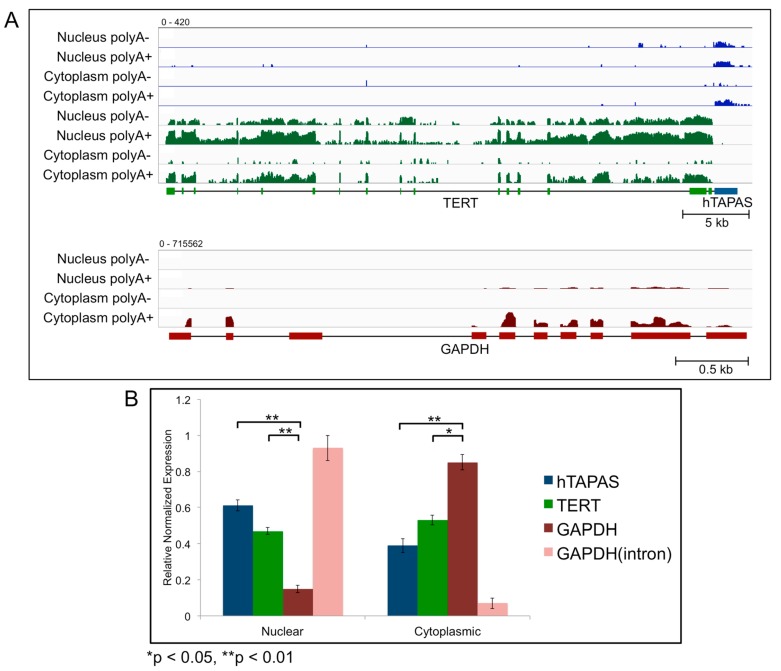
Sub-cellular localization of *hTERT* and *hTAPAS* RNAs (**A**) Normalized and stranded RNA-seq (Bedgraph) transcription coverage for *hTERT* (green) and *hTAPAS* (blue) expression are depicted for the polyadenylated (polyA+) and non-polyadenylated (polyA-) transcripts in the nuclear and cytoplasmic fractions, from the human B-cell cell line (GM12878). All plus strand (blue) and minus strand (green) track signals are depicted on a log-scale (0–420). Transcription coverage for corresponding transcripts of glyceraldehyde-3-phosphate dehydrogenase (*GAPDH*) (red) are depicted as a control, on a log scale (0–715562). Represented data are from the ENCODE Consortium. (**B**) Abundance of *hTAPAS*, *hTERT* and *GAPDH* (mRNA and pre-mRNA) transcripts in the nucleus and cytoplasm cellular fractions determined by quantitative Reverse Transcription PCR (qRT-PCR) after sub-cellular fractionation of HEK-293 cells.

To determine the sub-cellular localization of *hTAPAS* RNA, RNA-seq data was analyzed for polyadenylated (polyA+) and non-polyadenylated (polyA-) RNAs from cytoplasmic and nuclear fractions. We found evidence for both polyA+ and polyA- populations of *hTAPAS* in a human B-cell line (GM12878) (Figure 2A). The polyadenylated transcript was present in both nuclear and cytoplasmic fractions of the B-cell line. On the other hand, the polyA- transcript was absent from the cytoplasm. This suggests that a fraction of *hTAPAS* lncRNA is exported from the nucleus to the cytoplasm after being polyadenylated.

Surprisingly, a substantial fraction of the *hTERT* transcript was also observed in the nucleus of GM12878 cells (Figure 2A). A similar sub-cellular distribution of *hTERT* RNA was also seen in H1-hESC, HepG2, HeLa and K562 cell lines (data not shown). This was an unexpected observation since *hTERT* is a protein-coding gene and would be expected to be localized mostly in the cytoplasm. As expected, the control glyceraldehyde-3-phosphate dehydrogenase (*GAPDH*) mRNA was predominantly polyadenylated and cytoplasmic (Figure 2A).

We observed similar sub-cellular localization of *hTERT* and *hTAPAS* RNAs in both the nucleus and cytoplasm, after sub-cellular fractionation of HEK-293 cells and RNA quantitation by quantitative Reverse Transcription PCR (qRT-PCR) (Figure 2B). Again, GAPDH mRNA and pre-mRNA transcripts were mainly observed in the cytoplasmic and nuclear fractionations, respectively (Figure 2B). Further, we tested the possibility that *hTAPAS* RNA might be sequestering *hTERT* RNA in the nucleus. However, knocking down of *hTAPAS* via antisense oligonucleotides did not alter the cellular localization of *hTERT* in HEK-293 cells (data not shown). 

### 2.4. hTAPAS Expression Negatively Correlates with hTERT Expression in Cancer Patients 

**Figure 3:**
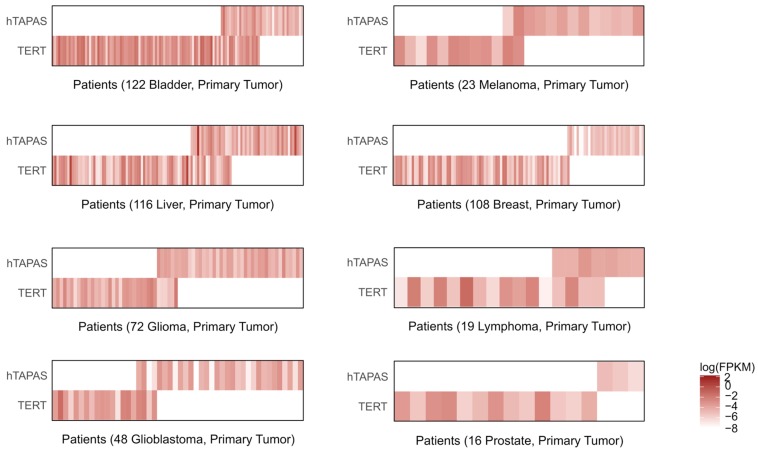
*hTERT* and *hTAPAS* expression are inversely correlated in primary tumors. RNA-seq data samples from TCGA, which have detectable expression levels for either *hTERT* or *hTAPAS* were analyzed in the eight different cancers (melanomas, gliomas, glioblastomas, hepatocellular carcinomas, bladder, lymphoma, prostate and breast cancers). A majority of these tumor samples express either *hTERT* or *hTAPAS*, but not both. The heat-maps depict corresponding expression levels of *hTAPAS* and *hTERT* among individual patient samples from the eight different cancers. Expression is represented as log-transformed Fragments Per Kilobase of transcript per Million mapped reads (FPKM) values (white indicates undetectable expression).

Antisense lncRNAs transcribed from bidirectional promoters have been known to be involved in regulation of the associated sense transcripts [21,22]. We thus investigated the relationship between the expression of *hTAPAS* and *hTERT* in TCGA database [23]. First, we analyzed *hTAPAS* and *hTERT* expression in primary tumor samples from eight different types of cancer by RNAseq (Table S1, Figure 3).

*hTERT* RNA detection ranged from 3 to 50% in the different types of primary tumors (Figure 3 and Figure S5A). Lymphomas had the highest percentage of patients expressing detectable levels of *hTERT*, followed by bladder and liver cancers, while prostate cancers had very few patients expressing detectable levels of *hTERT*. Tumor samples expressing detectable *hTAPAS* RNA ranged from 1% (prostate) to 20% (glioblastoma) (Figure S5B). In addition a few of the liver tumors expressed high levels of *hTAPAS* (Figure S3B). 

In the primary tumor samples in which at least one of these transcripts was detected, fewer than 20% expressed both *hTAPAS* and *hTERT* RNAs (Figure 3). Co-expression of *hTERT* and *hTAPAS* RNAs was nearly undetectable in breast and prostate cancers (Figure 3 and Figure S5C). The highest levels of co-expression (4–8%) of *hTERT* and *hTAPAS* RNAs were observed in primary tumors of lymphomas, bladder and liver cancers (Figure S5C). This pattern is suggestive of an antagonistic relationship between *hTAPAS* and *hTERT* expression. 

### 2.5. Knockdown of hTAPAS up Regulates hTERT Expression in HEK-293 Cells

**Figure 4:**
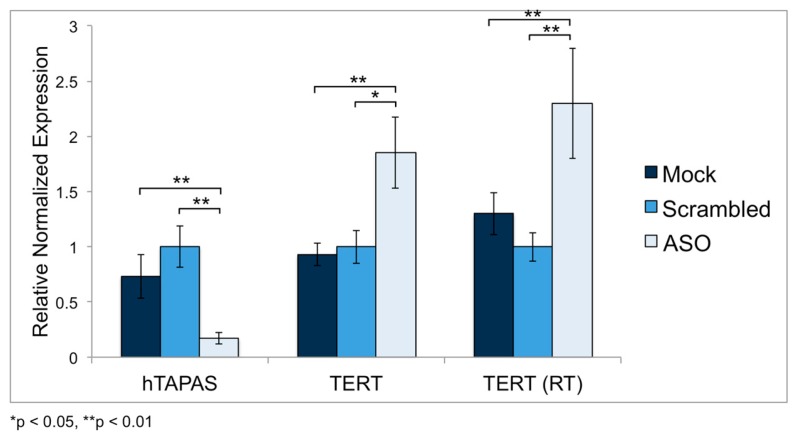
*hTAPAS* knockdown in HEK-293 cells results in increased *hTERT* expression. *hTERT* and *hTAPAS* expression was determined by qRT-PCR in HEK-293 cells transfected with mock, scrambled oligos, and antisense oligonucleotides (ASO) targeted to *hTAPAS*. Use of ASO resulted in a 4-fold knock down of *hTAPAS* expression, relative to scrambled. *hTERT* expression was analyzed using primers spanning its exons 13–14 or exons 7–8 (in the Reverse Transcriptase domain).

To test whether loss of *hTAPAS* RNA can alter *hTERT* expression, targeted knockdown of *hTAPAS* was performed in a HEK-293 cell line, using oligonucleotides antisense to the *hTAPAS* transcript (570 to 594 nts from the *hTAPAS* TSS). Cells transfected with a scrambled oligonucleotide showed no significant change in either *hTAPAS* or *hTERT* expression; however, transfection with an *hTAPAS* antisense oligonucleotide (ASO) resulted in a 4-fold knockdown of *hTAPAS* and a significant 1.8-fold increase in *hTERT* expression (using primers in *hTERT* exons 13 and 14) (Figure 4). We also measured expression of the *hTERT* transcript with primers in the catalytic reverse transcriptase domain (exons 7 and 8). Similarly, we detected a 2.1-fold increase in expression of these *hTERT* transcripts. These results suggest that *hTAPAS* lncRNA functions to negatively regulate *hTERT* expression.

### 2.6. hTAPAS Expression Is Not Affected by the hTERT Promoter Mutation

The mutant *hTERT* promoter can be bound and activated by GABP [15], suggesting it may induce bidirectional transcription of an antisense transcript [13,14]. Therefore, we asked whether this *hTERT* promoter mutation affects *hTAPAS* expression. We first analyzed RNA-seq data from eight hepatocellular carcinoma cell lines, four with and four without the *hTERT* promoter mutation -124 C>T (Figure S6) [6]. Consistent with previous reports [15], *hTERT* exhibited an average of approximately 2-fold higher expression in the cells with the promoter mutation, relative to the cells with the wild-type promoter (Figure S6). In contrast, no obvious difference in expression of *hTAPAS* was apparent between cell lines with the absence or presence of the point mutation (Figure S6). Furthermore, in cells with a wild-type *hTERT* promoter, *hTERT* and *hTAPAS* had similar expression levels; however, in cells with the presence of the point mutation, *hTERT* expression was approximately 2 to 4 fold higher than *hTAPAS* expression (Figure S6).

**Figure 5:**
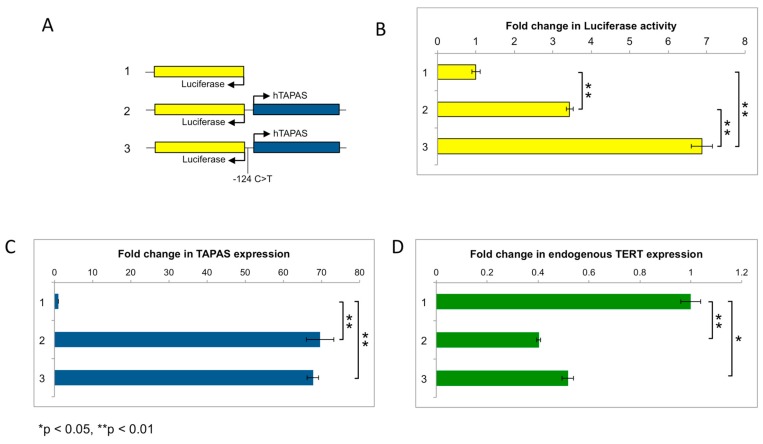
Presence of the *hTERT* promoter mutation does not alter *hTAPAS* expression. (**A**) Schematic representation of an empty control vector (1), and *hTAPAS* insert with a wild-type (2) or mutant (3) *hTERT* promoter region, in a luciferase reporter vector. (**B**) Fold-change in luciferase activity observed for the different constructs relative to empty vector. (**C**) Fold-change in *hTAPAS* or (**D**) endogenous *hTERT* expression levels observed for the different constructs relative to empty vector.

Next, we generated constructs to ectopically express the *hTERT* promoter region, with a luciferase reporter in lieu of *hTERT* (Figure 5A). We introduced the *hTERT* promoter point mutation (-124 C>T) into this construct to determine its influence on luciferase activity (as an indicator of *hTERT* expression) and *hTAPAS* expression in HEK-293 cells. We observed that luciferase activity was up regulated approximately 2-fold by the presence of the point mutation (Figure 5B), consistent with previously reported effects of the point mutation on *hTERT* expression. However, *hTAPAS* expression was not altered by the presence of the point mutation (Figure 5C). Moreover, in the ENCODE cell line HepG2, which has an *hTERT* promoter mutation, we observed *hTERT* expression but poor *hTAPAS* expression levels (Figure S1). Taken together, our data suggest that the *hTERT* promoter mutation significantly increases expression of *hTERT* but not of *hTAPAS*.

As a consequence of transfecting these luciferase constructs into HEK-293 cells, *hTAPAS* was ectopically over-expressed approximately 70-fold relative to endogenous levels (Figure 5C). *hTAPAS* over-expression resulted in down regulation of endogenous *hTERT* expression approximately 2-fold in HEK-293 cells. (Figure 5D). This expression pattern is consistent with our observations of *hTAPAS* knockdown resulting in increased *hTERT* expression levels (Figure 4). Therefore, these observations support our proposal that *hTAPAS* is involved in negatively regulating *hTERT* expression. 

## 3. Discussion

Telomerase activity in somatic cells is limited by the availability of *hTERT* protein and thus expression of *hTERT* is tightly regulated [24]. At the transcriptional level, *hTERT* is regulated by many transcription factors, such as c-MYC, ETS, and SP1/SP3, as well as through *hTERT* promoter mutations which can cause epigenetic modifications in the promoter region [7,25]. Extensive alternative splicing of the *hTERT* transcript has also been shown to generate inactive and dominant negative *hTERT* variants that decrease telomerase activity [6,7,8]. In this work, we identify a novel antisense lncRNA in the *hTERT* promoter region, *hTAPAS*, which functions as an additional negative regulator of *hTERT* expression. We also observed that a substantial portion of the *hTERT* transcript was localized in the nucleus, suggesting this might be an additional means of regulating the cellular abundance of catalytic *hTERT* by limiting its translation.

Many lncRNAs play a role in transcriptional regulation [26]. For example, *XIST* acts in *cis* to recruit the polycomb repressive complex 2 (PRC2) to chromosome X, causing gene silencing [27], while *HOTAIR* acts in *trans* to repress the expression of genes in the HoxD gene cluster [28]. It has been proposed that antisense lncRNAs transcribed from bidirectional promoters may be involved in regulation of their associated sense transcripts [21,28,29]. Such an arrangement may allow for tighter transcriptional regulation. We observed that *hTAPAS* RNA over-expression down-regulated *hTERT* expression in trans. We also observed that knocking down *hTAPAS* RNA up regulated *hTERT* RNA expression. This suggests a role of the *hTAPAS* RNA in the transcriptional regulation of *hTERT*. *hTAPAS* might recruit epigenetic machinery to regulate *hTERT* expression, similar to other well-studied lncRNAs [30]. In primary tumors in TCGA, we typically saw expression of either *hTERT* or *hTAPAS*, but not both. This suggests the possibility of promoter occlusion in the *hTERT* bidirectional promoter region. Furthermore, a substantial fraction of *hTAPAS* transcripts are exported to the cytoplasm. We cannot rule out its possible translation, as has been previously observed for other lncRNAs [31]. This suggests, that in addition to negative regulation of *hTERT* transcription, *hTAPAS* might be involved in other cellular functions in the nucleus and/or cytoplasm.

Shortening of telomeres limits the replicative potential of most primary human cells and serves a tumor-suppressive function. Conversely, telomerase expression in the germline, in somatic cells during early embryogenesis, and in cancer cells promotes telomere length homeostasis [2,32,33,34,35,36]. The preferential elongation of short telomeres and maintenance of telomere length homeostasis requires a low cellular concentration of telomerase [37]. This is achieved via expression of *hTERT* over a limited range, approximately 500 *hTERT* protein molecules per cell [37,38]. Telomere length regulation and telomerase expression in multicellular organisms is thought to have evolved by opposing selective pressures to suppress tumor formation on one side, while not promoting premature cellular senescence in highly proliferative tissues on the other side. Similar to *hTERT* expression, *hTAPAS* expression is observed in the germline, cancer cell lines and tumor samples, and is absent in normal tissues. Since *hTAPAS* is not expressed in normal tissues of cancer patients, it does not appear to be involved in negatively regulating *hTERT* expression in somatic cells. Therefore, it is possible that as a regulator of *hTERT* expression, *hTAPAS* might facilitate the maintenance of *hTERT* expression within the narrow range required for telomere length homeostasis in cancer cells and stem cells. 

We have previously reported the presence of numerous clonally expanded integrations of the avian leukosis virus (ALV) in the *TERT* promoter region in chicken B-cell lymphomas, associated with slightly elevated *TERT* expression [39,40]. We have also shown that proviral integrations in these tumors are in the antisense orientation relative to *TERT* and are driving the over-expression of chicken *TAPAS* lncRNA [16]. In this work we find evidence for an upstream antisense transcript in the *hTERT* promoter region. Similar to elevated expression levels of chicken *TAPAS* in B-cell lymphomas, we observe that *hTAPAS* also is expressed in immortalized human B-cell lines. Interestingly, the *hTERT* promoter region is also reported to be the most common integration site for hepatitis B virus integrations (HBV) in hepatocellular carcinomas [41,42,43,44]. Approximately 20% of these integrations are in the *hTERT* promoter region [41]. These integrations near *hTERT* are thought to confer an early clonal advantage during chronic HBV infection. Moreover, HBV integrations lead to up-regulated *hTERT* expression and are associated with poorer survival rates in infected patients with these integrations [42]. Of note, the frequency of *hTERT* promoter point mutations is significantly lower in hepatocellular carcinomas bearing HBV integrations in the *hTERT* promoter region [41].

**Figure 6:**
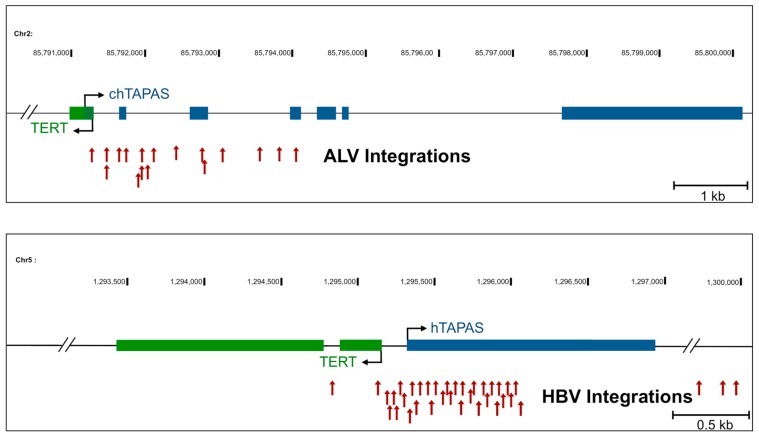
*TAPAS* is a viral integration hotspot in chicken and human tumors. The schematic represents the *hTERT* promoter region, including the transcription start sites (black arrows) for *hTERT* and *TAPAS*, in the chicken (top) and human (bottom) genomes. This region is depicted as a viral integration hotspot for the avian leukosis virus (ALV) and hepatitis B virus (HBV), respectively in tumors. Red arrowheads represent the integration sites for ALV or HBV in this region.

Interestingly, nearly all of the reported ALV and HBV integrations in the *hTERT* promoter region are present within the 5′ end of chicken *TAPAS* and *hTAPAS*, respectively (Figure 6). The majority of the ALV integrations in the chicken *TERT* promoter region are in the same transcriptional orientation as *TAPAS* [16]. In contrast, HBV integrations are in a mixed transcriptional orientation with respect to *hTAPAS* [41,42,43,44]. The prevalence of viral integrations within *TAPAS* in multiple types of cancers in different organisms suggests that these integrations are selected for during oncogenesis. These viral integrations might confer a proliferative advantage and make cells predisposed to oncogenic transformation. Since *hTAPAS* negatively regulates *hTERT* expression and HBV integrations would likely disrupt *hTAPAS* expression, this could promote *hTERT* expression. These observations, therefore, suggest functional implications of *hTAPAS* in tumorigenesis.

Like human telomerase, chicken telomerase is down regulated in most somatic tissues [45]. Furthermore, chicken telomeres shorten with age, and telomerase activity is important for oncogenesis [46]. In contrast, the mouse *TERT* enzyme is active in normal somatic cells [47]. This discrepancy between humans and mice is important because telomerase activation is a critical step in the human oncogenic process, with aberrant telomerase activation seen in most human cancers [3,4]. Further, no lncRNA expression has been observed in the *TERT* promoter region of mouse embryos (data not shown). This absence of a *TAPAS* transcript might facilitate increased *TERT* expression in mice. Therefore, chicken serves as an advantageous model to study oncogenic events of *TERT* signaling and transcriptional regulation.

LncRNAs, as well as bidirectional transcription, have arisen as novel players in tumorigenesis, warranting a need for further research of their role in regulating cancer development. The mutant *hTERT* promoter can be bound and activated by GABP, an ETS transcription factor [15], which has been shown to induce bidirectional transcription in other loci [13,14]. However, we observe no association of *hTAPAS* expression with the presence of a point mutation, in transfected HEK-293 cells or liver cancer cell lines. We observed that the most common point mutation in the *hTERT* promoter region (-124 C>T) does not affect *hTAPAS* expression. However, we did not test whether the -146 C>T point mutation affected *hTAPAS* expression. Additionally, we observe *hTAPAS* expression in the absence of detectable *hTERT* expression in some of the TCGA samples, but not in cell lines. This variation in the *hTAPAS* expression profile between primary tumor samples and cell lines could be due to the differences in the genomic features and microenvironments between cell lines and tumor samples [48,49]. 

Reactivated *hTERT* expression is reported in 90% of all human cancers [3,4]. However, through our analysis from the TCGA we detected *hTERT* expression in only 3–50% of analyzed samples. While previous studies have used qRT-PCR to observe *hTERT* transcript levels, our analysis involves the use of total RNA-seq data from primary tumors. The detection and quantification sensitivity of RNA-seq is dependent on the read depth. Even at an above average coverage of 100 million reads, RNA-seq suffers from greatly reduced quantification and detection sensitivity compared to qRT-PCR [50]. Therefore, the differences in our observations compared to previous work can be explained by the method used for detection of *hTERT* expression.

Activation of *hTERT* expression is a crucial step in the progression of many cancers, and understanding the molecular mechanisms of such activation is important for understanding oncogenesis. Recent genome-wide analyses have highlighted that somatic point mutations in the *hTERT* promoter are among the most common mutations in human cancer and are known to drive *hTERT* expression [51]. The *hTERT* promoter region is also the site of viral integrations in both chicken (ALV) and human (HBV) tumors (Figure 6). We observe that in many tumors there is activation of a previously uncharacterized antisense transcript in the *hTERT* promoter region, which we call *hTAPAS*. This lncRNA negatively regulates *hTERT* expression and, thus, is implicated in oncogenesis and telomere homeostasis.

## 4. Materials and Methods

### 4.1. Analysis of RNA Sequencing and CAGE Data 

Total and cellular fractionated RNA-seq data for analysis of expression of *hTAPAS* and *hTERT* in human cells were downloaded from the ENCODE Consortium and TCGA [17,23]. The ENCODE accession number for the total RNA-seq data analyzed for GM12878 is ENCSR889TRN, for OCI-LY7 is ENCSR001HHK, for H1-hESC is ENCSR537BCG, for HepG2 is ENCSR181ZGR, for HeLa-S3 is ENCSR552EGO, and for K562 is ENCSR000AEL. The RNA-seq data for sub-cellular fractions of GM12878 are available through the ENCODE RNA Dashboard (hg19) [17]. Data available through the ENCODE Data Coordination Center, and TCGA and represent normalized, stranded RNA-seq signal. RNA-Seq libraries for liver cancer cell lines were prepared in duplicate using the TruSeq stranded mRNA library kit according to manufacturers directions and sequenced on the Illumina HiSeq platform (San Diego, CA, USA). Two biological replicates were analyzed for each data set. The abundances of transcripts (fragments per kilobase per million, FPKM) were estimated and compared using Cufflinks [52]. CAGE data for analysis of *hTAPAS* and *hTERT* TSSs in GM12878 cells were downloaded from the ENCODE Consortium (ENCODE accession number ENCSR000CKA). 

### 4.2. Evolutionary Conservation and Coding Potential Analysis

phastCons, phyloCSF and phyloP scores, based on multi-way alignment of 20 mammalian genomes, were aggregated over the *hTAPAS* locus (chr5:1295329-1296947, 1619 positions), *hTERT* exons (NM_198253 exons annotated in hg38, 4,013 positions), and an intergenic sample of 10,000 positions from the *hTAPAS*/*hTERT* locus (chr5:1247464-1308783).

### 4.3. Fractionation Experiments

Sub-cellular fractionation of cultured HEK-293 cells was performed as follows. Cells were incubated in five volumes of isotonic lysis buffer and incubated on ice for 10 min. 1% NP40 was added with gentle inversion, followed by centrifugation at 5500 rcf for 30 s. The supernatant (cytoplasmic fraction) was removed into a separate tube. The pellet was re-suspended in five volumes of isotonic lysis buffer, followed by centrifugation at 1500 rcf for five min. The supernatant was added to the cytoplasmic fraction and the pellet was used as the nuclear fraction. 

### 4.4. RNA Isolation and Quantitative Reverse Transcription PCR

RNA from total cell and sub-cellular fractions was extracted using Trizol (Thermo Fisher, Scientific, Carlsbad, CA, USA). DNase I (Ambion) treated RNA was reverse transcribed with Maxima H minus reverse transcriptase (Thermo Fisher Scientific, Waltham, MA, USA) and oligo(dT)18 primer and/or random hexamers. *hTAPAS* was validated by a primer tiling array. All the PCR primers were commercially synthesized (Integrated DNA Technologies, Inc., San Diego, CA, USA). qRT-PCR was performed using iQ SYBR green Supermix (Thermo Fisher Scientific, Austin, TX, USA) on a Bio-Rad C1000 thermal cycler/CFX96 Real-Time System. The expression of *hTAPAS* RNA was measured using primers TGTAGCTGAGGTCGGCAAAC and GGTGCGAGGCCTGTTCAAAT. *hTERT* expression was measured using primers GTGCTGCAGCTCCCATTTCAT and GCTTTCAGGATGGAGTAGCAGA. Expression was normalized to the expression of GAPDH using exon junction primers AATCCCATCACCATCTTCCA and TGGACTCCACGACGTACTCA. qRT-PCR was performed in triplicate, with each sample present in technical duplicate during each run. The results were normalized using the comparative threshold cycle (C_T_) method.

### 4.5. hTAPAS Knockdown Experiments Using Antisense Oligonucleotides

ASO with phosphorothioate bonds were generated by Integrated DNA Technologies, Inc., San Diego, CA, USA. The ASO targeting *hTAPAS* binds 570 nts downstream from the transcription start site (GTGATTAACAGATTTGGGGTGGTTG). A scrambled ASO control was generated with matched base composition (GGTACGATTATTTCGGTTCGATTAGT). HEK-293 cells were transfected with 100 nM ASO using FuGene 6 (Promega, Madison, WI, USA) according to manufacturers protocol. Cells were then cultured for 48 h before being collected for measurement of RNA expression. *hTAPAS* primers flanked the ASO binding site (TGAGCAACCACCCCAAATCT, TTTCCCACCCTTTCTCGACG), *hTERT* primers spanned either the exon 13–14 junction (GTGCTGCAGCTCCCATTTCAT, GCTTTCAGGATGGAGTAGCAGA) or the exon 7–8 junction (GCGTAGGAAGACGTCGAAGA, ACAGTTCGTGGCTCACCTG). All expression was normalized to a housekeeping gene, GAPDH using primers AATCCCATCACCATCTTCCA and TGGACTCCACGACGTACTCA.

### 4.6. hTAPAS Constructs and Luciferase Assays

The *hTERT* promoter region, including *hTAPAS*, was cloned into the firefly luciferase construct pGL3-basic (Promega, Madison, WI, USA) using XhoI and Acc65I cloning sites. The *hTERT* promoter mutation (-124 C>T) was introduced by a quick-change PCR as described previously [53]. The pRL-CMV construct (Promega, Madison, WI, USA) was co-transfected into HEK-293 cells as a transfection control. 48 h after transfection, HEK-293 cells were harvested and assayed for firefly and Renilla luciferase activities by using the Dual-Luciferase Reporter Assay System (Promega).

### 4.7. Nucleotide Sequence Accession Number

The sequence of the *hTAPAS* cDNA, as validated by RT-PCR analysis, was submitted to GenBank under the accession number MG677549.

## Supplementary Materials

The following are available online at www.mdpi.com/2311-553X/4/1/1/s1, Table S1: Number of RNA-seq samples from TCGA for each cancer type investigated for *hTERT* and *hTAPAS* expression; Figure S1: Normalized and stranded RNA-seq (Bedgraph) transcription coverage for *hTERT* and *hTAPAS* RNAs for different ENCODE cell lines; Figure S2: Pipeline of TCGA RNA-seq data analysis; Figure S3: StringTie gene models, and expression of *hTAPAS* and *hTERT* in tumor and normal tissues of eight cancer types; Figure S4: *hTAPAS* does not exhibit conservation or protein-coding potential; Figure S5: Proportion of primary tumor samples with detectable expression of *hTERT*, *hTAPAS* or both; Figure S6: *hTAPAS* and *hTERT* expression in eight different human liver cancer cell lines, with the absence (−) or presence (+) of *hTERT* promoter mutation (-124 C>T).
