# Supplementary material for: A Novel Long Non-Coding RNA in the hTERT Promoter Region Regulates hTERT Expression
Source: Noncoding RNA. 2017 Dec 29;4(1):1. doi: 10.3390/ncrna4010001 (PMC5890388; doi:10.3390/ncrna4010001)
Supplement: Supplementary file 1 [file ncrna-04-00001-s001.pdf]

Cancer type	Metastatic	Normal Tissue	Primary Tumor	Recurrent Tumor
Bladder (BLCA)	0	**19**	**414**	0
Liver (LIHC)	0	**50**	**374**	0
Glioma (LGG)	0	0	**512**	**18**
Glioblastoma (GBM)	0	0	**156**	**13**
Breast (BRCA)	7	**123**	**1104**	0
Lymphoma (DLBC)	0	0	**48**	0
Prostate (PRAD)	1	**50**	**502**	0
Melanoma (SKCM)	**368**	0	**103**	0

Table S1. Number of RNA-seq samples from TCGA for each cancer type investigated for *hTERT* and *hTAPAS* expression.

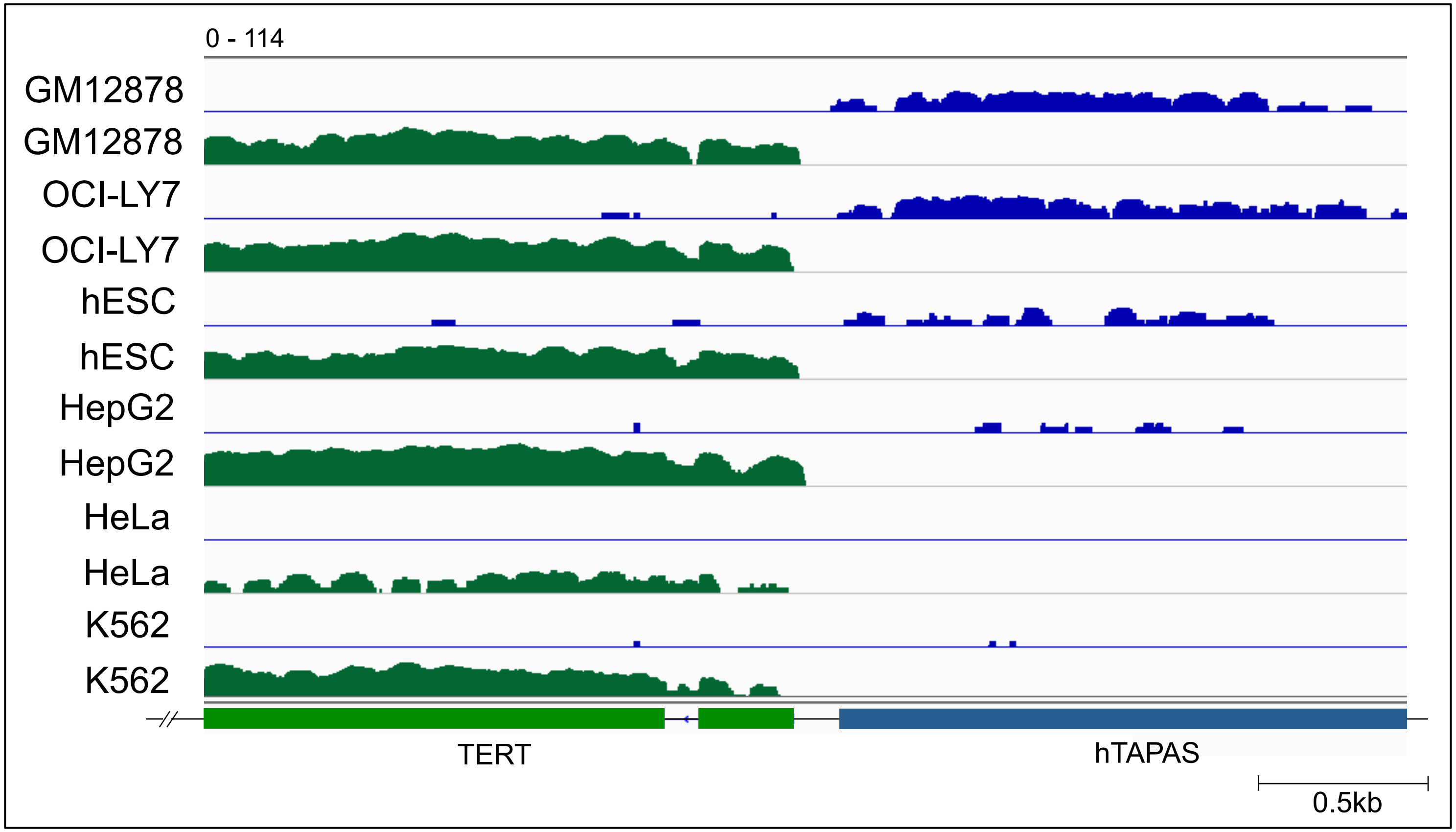

Figure S1. Normalized and stranded RNA-seq (Bedgraph) transcription coverage for *hTERT* (green) and *hTAPAS* (blue) RNAs are depicted for different ENCODE cell lines. All plus strand (blue) and minus strand (green) track signals are depicted on a log-scale (0 - 114). Represented data are from the ENCODE Consortium.

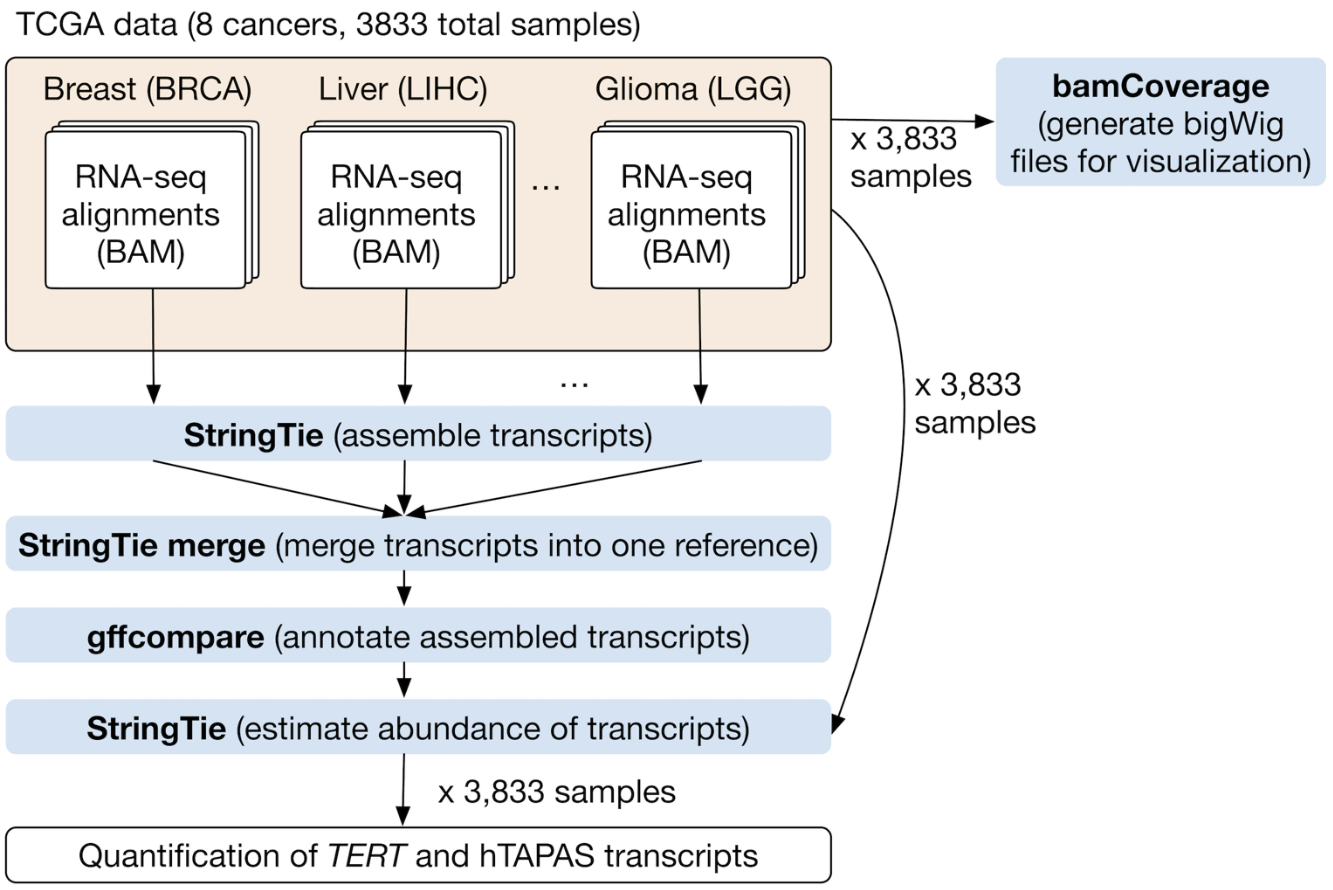

Figure S2. Pipeline of TCGA RNA-seq data analysis. Each STAR-aligned BAM file of RNA-seq data was downloaded for eight cancers (BRCA, BLCA, DLBC, GBM, LGG, LIHC, PRAD, SKCM) and used as input to StringTie without a reference transcript file to assemble transcripts. All assembled transcript files were merged using StringTie --merge with an hg38 reference GTF file of known transcripts to produce a single reference GTF file that was annotated using gffcompare. Then, each BAM file was used to quantify annotated transcripts using StringTie.

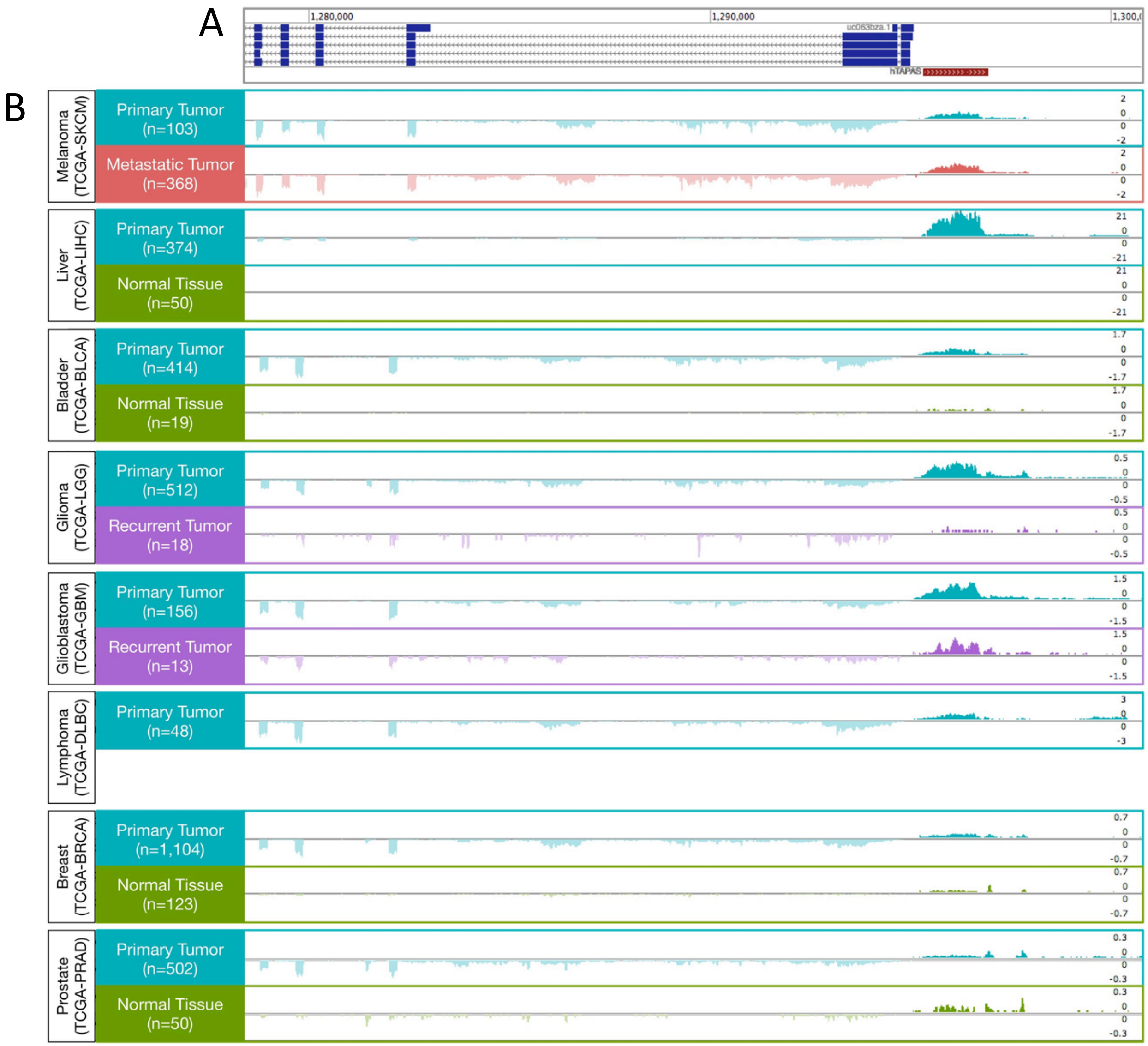

Figure S3. (A) Browser track with gene models shows UCSC *hTERT* gene models (dark blue) and StringTie-called *hTAPAS* gene model (red). (B) Expression of *hTAPAS* (plus strand, positive values) and *hTERT* (minus strand, negative values) in tumor and normal tissues of eight cancer types. Signal shown is FPKM summed over all samples.

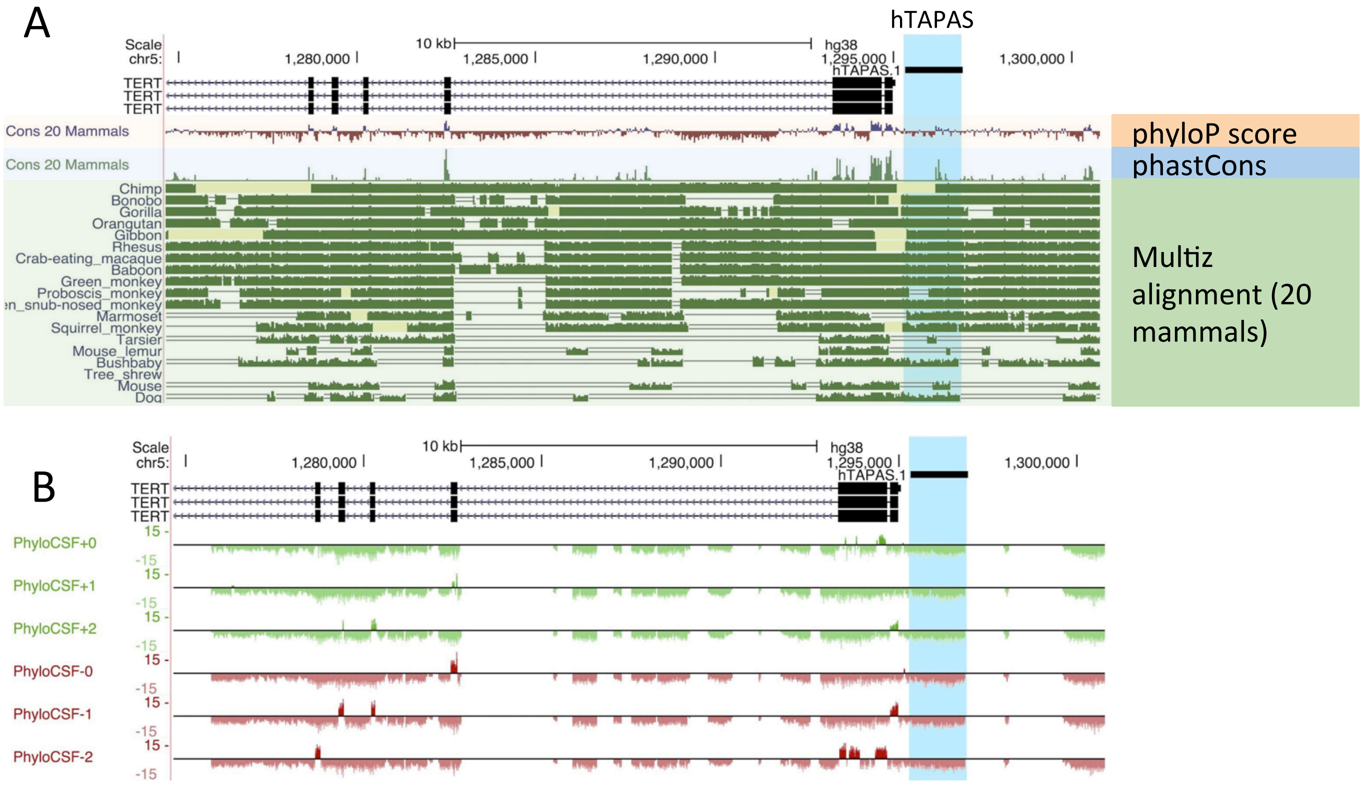

Figure S4. *hTAPAS* does not exhibit conservation or protein-coding potential. (A) PhyloP (top track) and phastCons (middle track) scores are displayed across the *hTERT* and putative *hTAPAS* genomic locus. Positive phyloP scores indicate conservation, while negative phyloP scores indicate faster rates of evolution compared to the neutral rate. A phastCons score of 0 indicates no conservation, while scores closer to 1 indicate higher conservation. Multiz alignment across 20 mammalian genomes (bottom track) suggest conservation among primates, but not other mammals. The *hTAPAS* locus displays more negative phyloP scores, indicating faster evolution rates than neutral, and lower phastCons scores, indicating low conservation, compared to *hTERT* exons. (B) PhyloCSF (codon substitution frequencies) scores based on alignment of 58 mammalian genomes are displayed for all six open reading frames across the *hTERT* and putative *hTAPAS* genomic locus. Positive values indicate that the region is likely to represent a conserved coding region, while negative values represent low protein-coding probability. *hTERT* exons overlap regions of positive phyloCSF scores, while the *hTAPAS* locus does not.

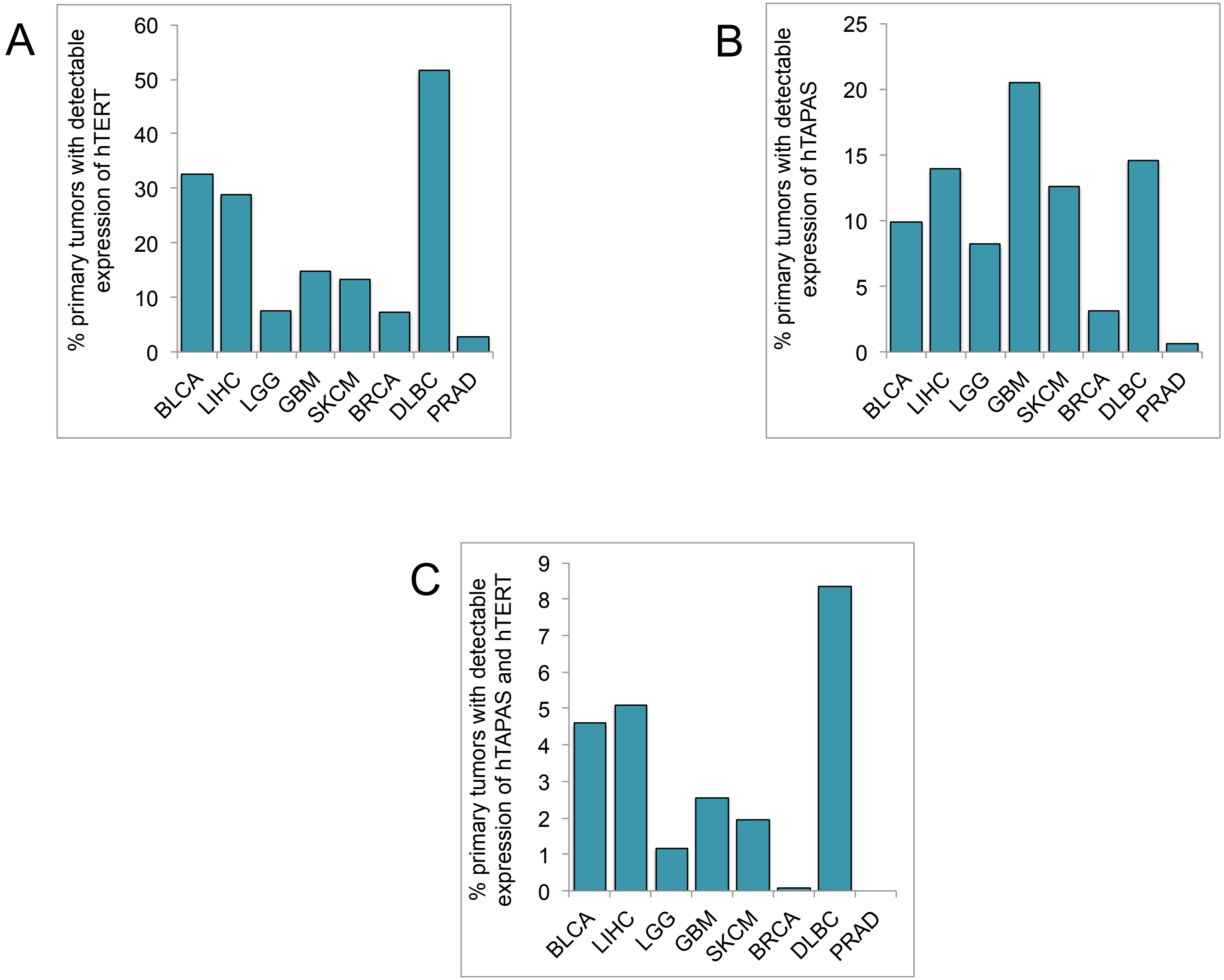

Figure S5. Proportion of primary tumor samples with detectable expression of (A) *hTERT* or (B) *hTAPAS* or (C) both, based on the TCGA RNA-seq data analysis.

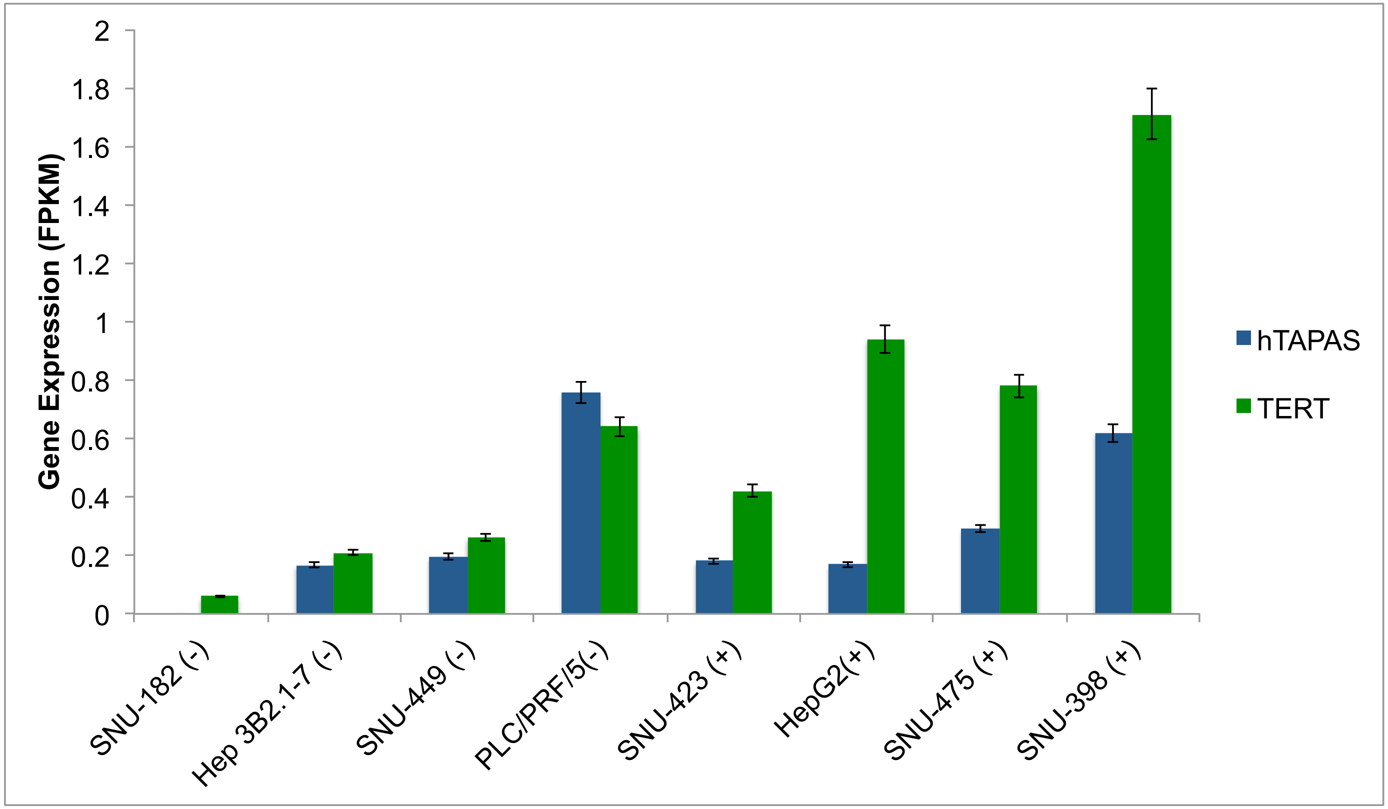

Figure S6. *hTAPAS* and *hTERT* expression (FPKM) in 8 different human liver cancer cell lines, with the absence (-) or presence (+) of *hTERT* promoter mutation (-66 nt from *hTERT* TSS).
